# Control of Signaling in a MAP-kinase Pathway by an RNA-Binding Protein

**DOI:** 10.1371/journal.pone.0000249

**Published:** 2007-02-28

**Authors:** Susanne Prinz, Christine Aldridge, Stephen A. Ramsey, R. James Taylor, Timothy Galitski

**Affiliations:** 1 Institute for Systems Biology, Seattle, Washington, United States of America; 2 University of British Columbia, Vancouver, British Columbia, Canada; Wellcome Trust Sanger Institute, United Kingdom

## Abstract

Signaling-protein mRNAs tend to have long untranslated regions (UTRs) containing binding sites for RNA-binding proteins regulating gene expression. Here we show that a PUF-family RNA-binding protein, Mpt5, represses the yeast MAP-kinase pathway controlling differentiation to the filamentous form. Mpt5 represses the protein levels of two pathway components, the Ste7 MAP-kinase kinase and the Tec1 transcriptional activator, and negatively regulates the kinase activity of the Kss1 MAP kinase. Moreover, Mpt5 specifically inhibits the output of the pathway in the absence of stimuli, and thereby prevents inappropriate cell differentiation. The results provide an example of what may be a genome-scale level of regulation at the interface of signaling networks and protein-RNA binding networks.

## Introduction

Mitogen-activated protein-kinase (MAPK) signal-transduction pathways function in biological responses ranging from yeast cell differentiation to mammalian immunity [1]. In diploid cells of the budding yeast, *Saccharomyces cerevisiae*, growth on low-ammonium solid media stimulates a signal-transduction network [2] mediating a switch from the ovoid single-cell yeast form of growth to a pathogen-like filamentous form characterized by cell elongation, unipolar distal budding to form cell chains, and substrate invasion [3]. A central module of this network is the filamentation MAP-kinase (fMAPK) pathway [4]. The fMAPK cascade includes the kinases Ste11, Ste7, and the Kss1 MAP kinase. Activated Kss1 derepresses the Ste12 Tec1 transcription-factor heterodimer [5], [6], which activates filamentation genes [7], [8] including *TEC1* itself.

The yeast *MPT5* gene, also known as *HTR1* [9], *UTH4* [10], and *PUF5* [11], encodes a Pumilio and *fem-3* binding (PUF) family RNA-binding protein required for multiple cellular processes. This is apparent in the pleiotropic phenotype of *mpt5* mutants. *MPT5* is required for longevity [10], [12], cell-wall integrity [13], [14], recovery from mating-pheromone arrest [15], [16], stress tolerance [9], [17], and post-transcriptional regulation of the *HO* gene [18]. The Mpt5 protein could affect these diverse processes through its ability to bind various mRNAs [19], or through its interactions with other proteins such as MAP kinases [15].

In the fruit fly, *Drosophila melanogaster*, the *pumilio* gene, a namesake of the PUF gene family, encodes a repressor of the translation of germ-line cell-differentiation proteins [20]. In yeast, links between *MPT5* and filamentous-form cell differentiation are suggested by network analyses [2], [4] integrating multiple data sources. In addition, we noticed in a genome-wide RNA-binding data set [19] that several of the 224 mRNAs bound by Mpt5 encode key filamentation signaling proteins. Based on these observations, we investigated a possible functional connection between Mpt5 and yeast filamentation. Here, we have established a major regulatory role for the Mpt5 RNA-binding protein in yeast cell differentiation. Specifically, we found that Mpt5 prevents inappropriate cell differentiation through the inhibition of fMAPK pathway activity.

## Results

**Figure 1:**
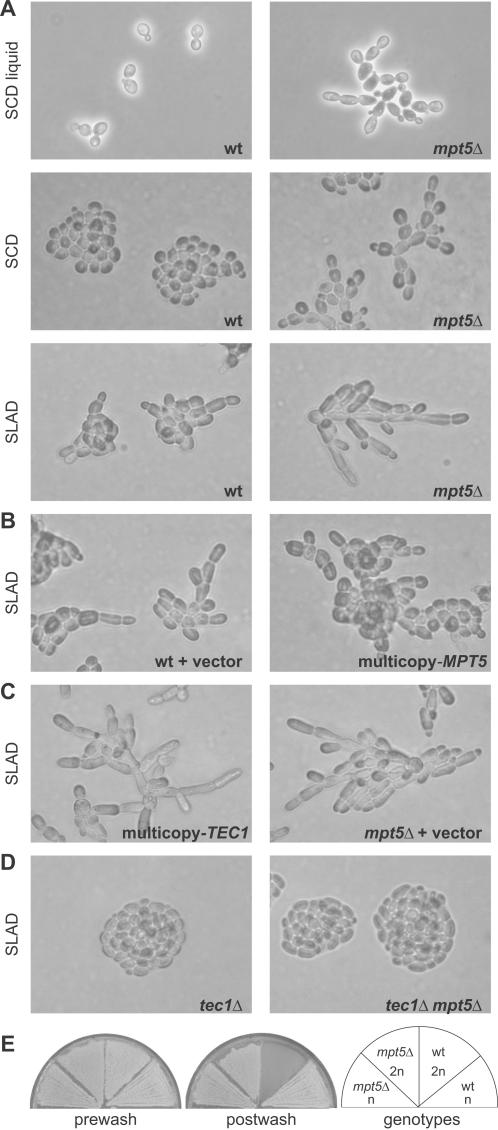
Repression of yeast filamentous-form phenotypes by *MPT5*. (A–D) Diploid yeast were grown under yeast-form conditions (SCD liquid and SCD) or filamentous-form conditions (SLAD) and microscopically imaged. (E) Patches of yeast were grown on rich medium agar and subjected to a washing-off assay of adhesion.

We found that the *MPT5* gene encodes a repressor of yeast cell differentiation to the filamentous form. Deletion of *MPT5* from filamentation-competent diploid yeast results in a constitutively filamentous phenotype. Mutant *mpt5*Δ yeast filament in the absence of filamentation stimuli, and are hyper-filamentous under filamentous-form growth conditions (Fig. 1A). Overexpression of the *MPT5*+ gene on a multicopy plasmid (Materials and Methods) antagonizes filamentation under filamentous-form growth conditions (Fig. 1B). The striking phenotypes of these mutants suggest that *MPT5* may exert its effects through regulation of the fMAPK pathway. Supporting this suggestion, the *mpt5*Δ phenotype resembles the multicopy *TEC1*+ phenotype (Fig. 1C) and requires an intact *TEC1* gene (Fig. 1D).

*MPT5* is also a repressor of adhesion, a filamentation-associated process. Haploid yeast cells adhere to and invade an agar surface when grown for an extended time in patches on rich medium [21]. The fMAPK pathway activates this adhesion [21], which is repressed in diploids by the mating-type genes and by increased ploidy [22]. In an assay of resistance to washing off a rich-medium agar surface (Materials and Methods), wild-type diploids wash off readily whereas *mpt5*Δ diploids adhere avidly (Fig. 1E); haploids serve as positive controls. Thus, deletion of *MPT5* derepresses two fMAPK-related phenotypes, filamentous-form growth and adhesion.

**Figure 2:**
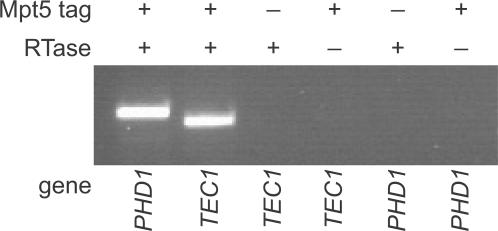
**In vivo** *TEC1* mRNA binding by Mpt5. An immunoprecipitate of epitope-tagged Mpt5 protein was subjected to reverse transcription and gene-specific polymerase chain reaction to detect the presence of bound mRNAs. Control experiments lacked either the epitope tag or reverse transcriptase.

Mpt5, a PUF protein, binds to the mRNAs of several filamentation signaling genes, including *PHD1, RAS2*, and *STE7* [19]. The epistasis of *tec1*Δ to *mpt5*Δ (Fig. 1D), and the strong resemblance of the *mpt5*Δ and multicopy *TEC1*+ phenotypes (Figs. 1A and C), led us to investigate the possibility of a link of *MPT5* with *TEC1*. We found that the *TEC1* mRNA immunoprecipitates with Mpt5 protein *in vivo* (Fig. 2). We appended a 13-myc epitope tag to the endogenous *MPT5* gene. Tagged Mpt5 protein was immunoprecipitated from diploid yeast cells (Text S1). To detect mRNAs, the immunoprecipitate was subjected to reverse transcription and polymerase chain reaction. *PHD1* served as a positive control. Negative-control experiments lacking either reverse transcriptase or the 13-myc tag assured that the detected sequences were neither DNA nor unbound co-purifying mRNA. No other fMAPK pathway components were tested; it remains possible that the mRNAs of other components are bound by Mpt5.

The results suggest that the repression of yeast cell differentiation by the Mpt5 protein is due to effects on the fMAPK pathway. Nonetheless, the repression of filamentation by *MPT5* might involve the binding of the Mpt5 protein to the mRNAs of major regulators of filamentation that are outside the fMAPK pathway [19], notably Phd1, a transcription factor whose overexpression induces filamentous growth [23], and Ras2, a GTPase whose activation stimulates filamentation by activating the fMAPK and cyclic-AMP/protein-kinase-A pathways [3]. However, in contrast with the requirement for an intact *TEC1* gene (Fig. 1D), the *mpt5*Δ mutant phenotype requires neither *PHD1* nor *RAS2* (Fig. S1). Thus, the fMAPK pathway is a major mediator of the control of yeast cell differentiation by *MPT5*.

**Figure 3:**
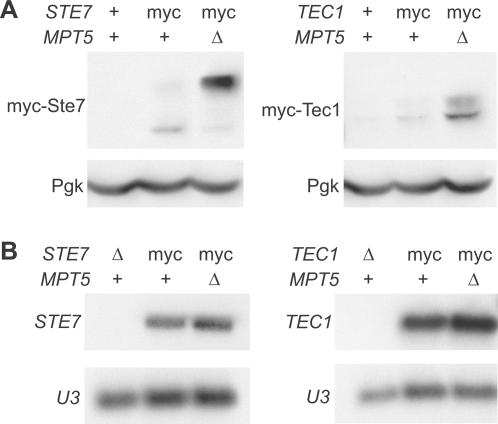
Repression of Ste7 and Tec1 protein levels by *MPT5*. (A) Yeast strains were grown under yeast-form conditions. Protein extracts were analyzed by western blot, with Pgk serving as a loading control. (B) RNA extracts were analyzed by northern blot, with *U3* serving as a loading control.

The interaction of the Mpt5 protein with the *STE7* and *TEC1* mRNAs, combined with the molecular activity of PUF proteins as translational repressors [24], [25] and mRNA de-adenylation factors [26], raises the possibilities that Mpt5 represses Ste7 and Tec1 protein expression or that Mpt5 destabilizes the mRNAs of these proteins. To test these possibilities, we constructed (Text S1) diploid strains with triple-myc epitope tags on the 5′ ends of the endogenous *STE7* and *TEC1* coding sequences. The modified genes are under the control of their native promoters, terminators, and UTRs. *MPT5*+ and *mpt5*Δ strain pairs were constructed. Protein and total-RNA extracts were prepared from cultures grown under yeast-form conditions, and were subjected to western-blot (Fig. 3A) and northern-blot (Fig. 3B) analyses. *MPT5* represses Ste7 and Tec1 protein levels (Fig. 3A), and has a minor (Fig. 3B) but reproducible (data not shown) negative effect on *STE7* and *TEC1* mRNA levels. These results suggest that the Mpt5 protein represses Ste7 and Tec1 protein levels primarily at the level of protein translation from their respective mRNAs.

Note also that loss of *MPT5* activity results in an increase in low-mobility forms of the Ste7 and Tec1 proteins (Fig. 3A). For Ste7, nearly all of the protein is in the low-mobility form. These low-mobility forms are phosphorylated protein. Treatment with phosphatase converts them to high-mobility forms (Fig. S2; Text S1).

**Figure 4:**
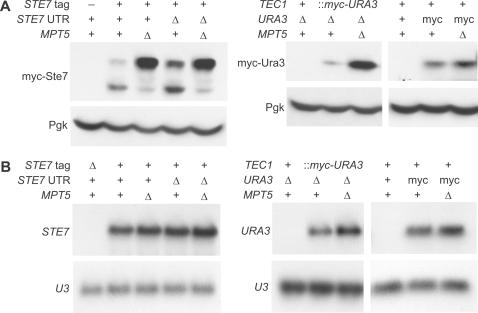
UTR sequences and repression by *MPT5*. Yeast strains were grown under yeast-form conditions. Protein and RNA extracts were prepared and analyzed by (A) western blot and (B) northern blot. Pgk protein and *U3* RNA served as loading controls.

Mpt5 and other PUF proteins are known to bind to sequence motifs in the 3′ untranslated regions (3′ UTR) of mRNAs [18], [19], [27]. Gerber *et al*. [19] have identified an 11-base sequence motif in 3′ UTRs of 33% of the mRNAs bound by the Mpt5 protein. We mapped the 3′ ends of the *STE7* and *TEC1* mRNAs (Text S1). The *STE7* and *TEC1* 3′ UTRs extend 133 and 107 bases past their respective stop codons (data not shown), and thus are above the median 3′UTR length of 91 nucleotides [28]. Within the *STE7* 3′ UTR, there is a single site (AUGUAACAAUA, starting at the 119th base after the stop codon) matching the Mpt5 RNA-binding motif. We found that precise deletion [29] of this site results in derepression of Ste7 protein levels in an *MPT5*+ strain (Fig. 4A). Also, deletion of this site results in a partial increase in Ste7 phosphorylation (Fig. 4A). The combination of the binding-site-sequence deletion and deletion of *MPT5* results in both derepression of Ste7 protein levels and maximum Ste7 phosphorylation (Fig. 4A).

Like 67% of the mRNAs bound by Mpt5 [19], the *TEC1* 3′ UTR (and the 5′ UTR) contains no match to the Mpt5 binding-site motif. Mpt5 presumably binds either directly or indirectly to some other sequence in these mRNAs. Alternatively, the binding-site motif may require refinement. We tested the hypothesis that repression of Tec1 protein levels involves its UTR sequences. We replaced myc-Tec1 protein-coding sequences with myc-Ura3 protein-coding sequences (Text S1). In the mRNA expressed from this hybrid *TEC1*::*myc-URA3* gene, *TEC1* UTR sequences flank myc-Ura3 protein-coding sequences. As observed for the *TEC1* gene encoding myc-Tec1 protein (Fig. 3A), *MPT5* represses the levels of myc-Ura3 protein translated from an mRNA with *TEC1* UTRs (Fig. 4A), and exerts a minor negative effect on the levels of the Ura3-encoding mRNA (Fig. 4B). Thus, the effect of *MPT5* on Tec1 protein levels is independent of Tec1-protein sequences and *TEC1*-protein-coding nucleic-acid sequences. As a control, we tested the effect of *MPT5* deletion on the levels of myc-Ura3 protein expressed from the *URA3* gene and mRNA (with *URA3* UTRs) (Fig. 4). The results show that *MPT5*-dependent repression of myc-Ura3 expression is imparted by *TEC1* UTR sequences but not *URA3* UTR sequences. We conclude that direct or indirect interaction of Mpt5 protein with UTR sequences mediates repression of Tec1 protein levels.

**Figure 5:**
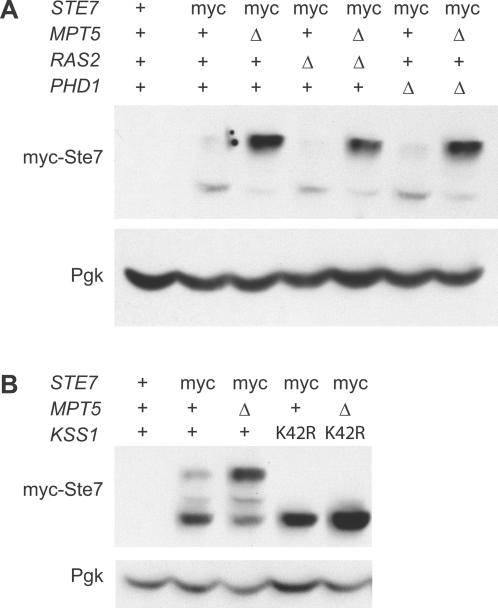
Inhibition of Kss1-dependent phosophorylation of Ste7 by *MPT5*. Yeast strains were grown under yeast-form conditions. Protein extracts were analyzed by western blot, with Pgk serving as a loading control. The effect of *MPT5* deletion on Ste7 phosphorylation is independent of *RAS2* and *PHD1* (A) but depends on *KSS1* (B).

Deletion of the Mpt5 binding sequence in the *STE7* message results not only in derepression of Ste7 protein levels, but also in an increase of Ste7 phosphorylation. However, only when *MPT5* is deleted is Ste7 maximally phosphorylated (Fig. 4A). These observations suggest *MPT5* has an effect on Ste7 phosphorylation through a mechanism that is separate from its effect on Ste7 protein levels. The effect on Ste7 phosphorylation depends on neither *RAS2* nor *PHD1* (Fig. 5A). However, we found that maximal phosphorylation of Ste7 depends entirely on Kss1. We constructed strains that harbored the kinase-dead allele *kss1K42R* [5] and were either *MPT5*+ or *mpt5*Δ. Extracts were prepared from cultures grown under yeast-form conditions, and were subjected to western-blot analysis detecting myc-Ste7. Whereas Ste7 protein levels are higher in *mpt5*Δ strains regardless of the *KSS1* allele, accumulation of maximally phosphorylated forms of Ste7 in *mpt5*Δ strains requires the kinase activity of Kss1 (Fig 5B). These results suggest that Mpt5 represses Ste7 and Tec1 protein levels, and in addition exerts a negative effect on Kss1 kinase activity.

**Figure 6:**
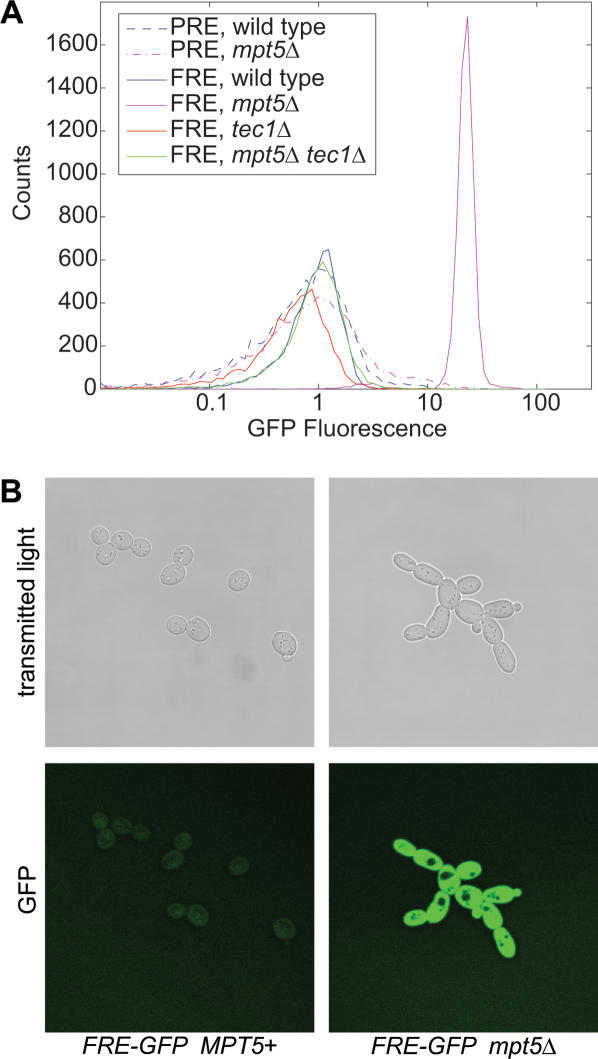
Specific inhibition of fMAPK pathway output by *MPT5*. (A) Diploid strains with either a minimal filamentation-MAPK-pathway output reporter (FRE-GFP) or a mating-MAPK reporter (PRE-GFP) and the indicated genotypes were grown under yeast-form conditions and subjected to flow cytofluorometry. (B) The morphology and fluorescence of *MPT5*+ and *mpt5*Δ diploid cells with FRE-GFP were imaged by transmitted light and confocal fluorescence microscopy.

The genetics and molecular biology of the *MPT5* gene suggest that it encodes an inhibitor of fMAPK pathway signaling. We tested this suggestion directly. fMAPK signaling derepresses the Ste12-Tec1 heterodimer, which binds to the Filamentation Response Element (FRE) in filamentation-gene promoters [7]. We fused a minimal FRE-dependent promoter [7] to Green Fluorescent Protein (GFP) coding sequences and integrated this fMAPK-pathway output reporter in the genome (Text S1). This diploid strain, plus *mpt5*Δ, *tec1*Δ, and *mpt5*Δ *tec1*Δ mutant derivatives, were grown under yeast-form conditions and analyzed by flow cytofluorometry (Materials and Methods). Deletion of *MPT5* derepresses the fMAPK output reporter by 16-fold in the absence of pathway stimuli (Fig. 6). This effect requires *TEC1* (Fig. 6A) and is accompanied by filamentous cell morphology (Fig. 6B). We conclude that Mpt5 is an inhibitor of signaling in the fMAPK pathway.

The fMAPK pathway shares multiple components, including Ste7 and Ste12, with the mating MAP-kinase (mMAPK) pathway, which is inactive in diploids. Because deletion of *MPT5* activates the fMAPK pathway in the absence of stimuli, it was possible that it also results in inappropriate activation of mMAPK pathway output in diploids. We tested this possibility. In the GFP-based pathway reporter, FRE sequences were replaced with the triple Pheromone Response Element (PRE) sequences of the *PRM1* promoter [30]. Though this reporter is induced sharply by pheromone in haploids (Fig. S3), it shows essentially no response to deletion of *MPT5* in diploids (Figure 6A), and haploids (Fig. S3). Thus, Mpt5 inhibits signaling in the fMAPK pathway specifically.

## Discussion

The combination of the amplification effect of MAPK cascades [31] and random noise, a substantial feature of signaling pathways [32], creates the risk of pathway off-state instability. This observation suggests the existence of mechanisms to prevent pathway output in the absence of a pathway stimulus. Our work suggests that Mpt5 serves this purpose in the fMAPK pathway and thereby prevents inappropriate filamentous-form differentiation.

We have shown that Mpt5 represses the protein levels of the MAP-kinase kinase Ste7 and the downstream transcription factor Tec1, and that this regulation is mediated by UTR sequences in their respective mRNAs. These effects may limit the signal capacity of the fMAPK pathway and thereby stabilize the pathway ‘off’ state. Furthermore, Mpt5 negatively regulates Kss1-dependent phosphorylation of Ste7. Kss1 phosphorylates Ste7 in vitro [33]. Thus, the simplest model to explain the effect on Ste7 phosphorylation is that Mpt5 negatively regulates the kinase activity of Kss1. At present, it is unclear whether the effect on Kss1 is direct or indirect. Because the Mpt5 and Kss1 proteins interact in a two-hybrid assay [15], a direct effect is possible. Also requiring further study is the question of how the activity of Mpt5 is regulated. Mpt5 protein abundance, as determined by western-blot analysis, does not differ in yeast-form cells and constitutively filamentous *STE11-4* cells (S. Prinz, unpublished). Changes in the subcellular localization of Mpt5 or its target mRNAs could provide a regulatory mechanism. Also, the physical interaction of Mpt5 with the Kss1, Fus3, and Cdc28 kinases [15] raises the possibility that Mpt5 is a kinase substrate regulated by phosphorylation.

Our work may indicate a positive feedback operating in the fMAPK pathway. This is suggested by the observation that the increase of Ste7 protein due to the deletion of the Mpt5 binding site results also in increased Kss1-dependent phosphorylation of Ste7 protein. Though the functional consequences of Ste7 phosphorylation by Kss1 are not clear, Maleri et al. [34] have shown that it does not result in attenuation, as had been assumed previously [35]. Previous studies have shown maximal phosphorylation of Ste7 to be dependent on pheromone stimulation and Kss1 and Fus3 activity [36], [37]. Similarly, we found the phosphorylation state of Ste7 to be indicative of the activity of the fMAPK pathway. In mutants that are constitutively filamentous such as *mpt5Δ* (Fig. 3A) or *STE11-4* [36] (data not shown), Ste7 is maximally phosphorylated, whereas in yeast-form cells (Fig. 3) or kinase-dead *kss1K42R* mutants (Fig. 4B) Ste7 is mainly unphosphorylated. Thus, we speculate that Kss1 acts on Ste7 in a positive feedback promoting the switch to the pathway ‘on’ state. Negative regulation by Mpt5 could stifle this effect and stabilize the ‘off’ state. Further molecular genetics, in conjunction with pathway modeling studies, could test these possibilities.

In conclusion, the combination of the ubiquity of RNA-binding proteins, the long binding-site-containing UTRs of mRNAs encoding regulatory proteins [19], [28], [38], and the emerging role of RNA-binding proteins in signaling [39], [40], suggests that there is a genome-scale level of regulation to be studied. Post-transcriptional control of gene activity by RNA-binding proteins may be critical for tight regulation of signaling and, consequently, the control of cell differentiation. Our results establish an example.

## Materials and Methods

### Growth conditions

Standard media and conditions were used for yeast-form [41] and filamentous-form [3] growth. Tests of filamentation and adhesion were done as described previously [2].

### Immunoprecipitation and RT-PCR

Immunoprecipitation of Mpt5-13myc and bound RNAs from yeast-form cells with ensuing RT-PCR was done as described [40], except for the following: protease inhibitors were added as in Ren *et al*. [42]; the antibody was mouse anti-myc monoclonal 9E10 (Covance); beads were ImmunoPure Immobilized Protein G beads (Pierce); incubation with beads was for 2 hours; 1st strand cDNA synthesis was primed with a polyT oligonucleotide. Gene-specific PCR amplification used the following primers: TEC1_RACE (GAAAGTAATCCTGAGTT CAGTTCCA); TEC1_3UTR_R (GTTCGAGAACTGGTAATGTTTGACT); PHD1_RACE (AAGTTGTTGAATGTCACGAAGATG); and PHD1_3UTR_R (ATTG TACGAATCCTATCAGCCTTTC).

### Protein extracts and western-blot analyses

Protein-extract preparations and western-blot analyses were done as described previously [2], except that extracts included Phosphatase Inhibitor Cocktails 1 and 2 (Sigma). The myc epitope was detected using mouse monoclonal antibody 9E10 (Covance). High-Range Rainbow (GE Healthcare) molecular weight markers were used. Triple-myc epitope-tagged Ste7 and Tec1 proteins showed gel mobilities corresponding to estimated molecular weights of 72 kD and 73 kD, respectively. The mobilities of phosphorylated Ste7 and Tec1 correspond to apparent molecular weights of approximately 84 kD and 81 kD.

### RNA preparation and northern-blot analyses

RNA-extract preparations and northern-blot analyses were done as described previously [2]. *TEC1* and *STE7* probes were made by PCR using primers: TEC1_C_F (AAGTGCGTTCCGTCAAAGAG), TEC1_C_R (ATTGGTTGGATGGCGTAAAG ), Ste7_probe_F (TCTTAACCTGCAC CCAGATG), Ste7_probe_R (ATTTGAAGTTCCCGACAACG). Primers for the *URA3* probe are described in Text S1. The *U3* (*SNR17A*) probe was made using primers described elsewhere [43].

### GFP fluorescence measurement and imaging

Exponential-phase cells grown in SCD medium were inspected and imaged with transmitted light and fluorescence using a Leica confocal microscope. For flow cytofluorometry, cells were briefly sonicated to disrupt cell aggregates. GFP fluorescence was measured using a Becton Dickinson FACSCalibur flow cytometer. Wild-type and *mpt5*Δ strains lacking GFP were included as autofluorescence controls. The scatter plots of fluorescence intensity (FLI) and forward light scatter (FLS) for the autofluorescence control strains were used to fit a function of FLI versus FLS using least squares. The control strains' FLS data were used to calculate FLS window sizes using the overall standard deviation of FLS. To ensure a homogeneous population of events and remove outliers (multi-cell events, etc.), this window was applied to the flow cytometry data for each strain, selecting only those events within the FLS window centred at each strain's mean FLS. The cell-size-dependent bias in FLI data was corrected by subtracting the FLI-versus-FLS function from the fluorescence of each event based on the event's FLS value. An additive correction was applied to ensure that the mean background-subtracted FLI for events within the window is the same as the raw (uncorrected) FLI for events within the window. For each event within the window, the background-subtracted FLI and the FLS were normalized relative to the overall mean FLI and FLS of the control strain, respectively. The average FLS ratios for the four strains were: 1.05 (wild type), 1.02 (*tec1*), 1.45 (*mpt5*), and 1.22 (*mpt5 tec1*). Background subtraction did not change the median FLI, but reduced cell-to-cell variation in FLI by approximately 5%.

## Supporting Information

Text S1 Supporting Materials and Methods
(0.14 MB PDF)

Figure S1 *mpt5Δ* phenotype requires neither *PHD1* nor *RAS2*. Diploid yeast of the indicated genotypes were grown under filamentous-form conditions (SLAD agar) and microscopically imaged to show their filamentation phenotypes.
(0.93 MB PDF)

Figure S2 Phosphorylation of Tec1 and Ste7 in *mpt5Δ* strains. N-terminally tagged Tec1 or Ste7 protein, or a tagless control, was immunoprecipitated from an *mpt5Δ* strain with subsequent phosphatase treatment or mock treatment and analyzed by western blot.
(0.92 MB PDF)

Figure S3 fMAPK and mMAPK pathway output in haploids. Haploid *MAT*a strains with either a minimal filamentation-MAPK-pathway output reporter (FRE-GFP) or a mating-MAPK reporter (PRE-GFP) and the indicated genotypes were grown under yeast-form conditions in the absence of alpha factor and subjected to flow cytofluorometry. As a control, PRE-GFP output of an alpha-factor stimulated strain is shown.
(0.52 MB PDF)
