# Supplementary material for: Control of Signaling in a MAP-kinase Pathway by an RNA-Binding Protein
Source: PLoS One. 2007 Feb 28;2(2):e249. doi: 10.1371/journal.pone.0000249 (PMC1803019; doi:10.1371/journal.pone.0000249)
Supplement: Text S1 — Supporting Materials and Methods (0.14 MB PDF) [file pone.0000249.s001.pdf]

SUPPORTING MATERIALS AND METHODS

Control of signaling in a MAP-kinase pathway by an RNA-binding protein

Susanne Prinz[1], Christine Aldridge[1]†, Stephen A. Ramsey[1], R. James Taylor[1,2], and Timothy Galitski[1,2]

[1]Institute for Systems Biology, 1441 N. 34th Street, Seattle, WA 98103, USA. [2]University of British Columbia, Vancouver, BC V6T 1Z4, Canada

†Present address: Institute for Cell and Molecular Biosciences, University of Newcastle, Newcastle upon Tyne NE2 4HH, UK

Correspondence and requests for materials should be addressed to T.G. (tgalitski@systemsbiology.org).

This project was supported by grant P50 GM076547 from NIH. S.A. Ramsey was supported by grant U54 AI054523R from NIH. R.J. Taylor was supported by a junior graduate studentship from the Michael Smith Foundation for Health Research. T. Galitski is a recipient of a Burroughs Wellcome Fund Career Award in the Biomedical Sciences.

Strain constructions. Strain G85, a Σ1278b *MAT***a**/*α ura3Δ0/ura3Δ0 his3Δ0::hisG/his3Δ0::hisG* diploid, served as the wild type and the parent of all yeast strains in the study. Standard methods [1] were used for all transformations and crosses to construct homozygous diploid mutant derivatives. The *TEC1*, *PHD1*, and *RAS2* deletion-insertion alleles, *tec1*Δ::bcKanMX4, *phd1*Δ::bcKanMX4, and *ras2*Δ::bcKanMX4, respectively, were constructed by Drees *et al*. [2]. These alleles are referred to as '*tec1*Δ', '*phd1*Δ', and '*ras2*Δ' in the text. The *ste7*Δ strain, from the yeast "barcode" deletion collection, was obtained from Research Genetics. Constructions of genomic insertion and deletion-insertion alleles used PCR-based methods and transformation with appropriate selection. All allele constructions were verified by PCR and tetrad dissection. All protein epitope tags were verified additionally by western-blot analysis and phenotype testing. All alleles expressing tagged proteins were functional. To delete the *MPT5* gene, resulting in the *mpt5*Δ::kanMX6 allele ('*mpt5*Δ'), plasmid pFA6a-kanMX6 [3] was the PCR template with primers MPT5F1 (CTACGCAAATTTATAAATCAATTACGATTTTTCCAGTTTCTCTTCGGATCCCCGGGTTAATTAA) and MPT5R1 (TCCGGCTCAAATTTTGGGACTTTAGTAGGACGTCATTGCTTAGTGAATTCGAGCTCGTTTAAAC). The Mpt5 protein was tagged at its C-terminus with the 13-myc epitope using pFA6a-13Myc-kanMX6 [3]. Using a PCR-based two-step tagging method [4], Tec1 and Ste7 proteins were tagged at their N-termini with a triple-myc epitope tag, leaving the native promoters and UTRs intact. This *myc-TEC1* construct was converted to a *TEC1*::*myc-URA3* construct (myc-Ura3 protein-coding sequences replacing myc-Tec1 coding sequences at the TEC1 gene). Using plasmid pRS406 [5] as a template with primers (*URA3* priming sequences underlined) 3myc-URA3-F (GCCGCACTAGTCTCGAGGGGGGGCCCGGTACCCAATTCGCCCTACAGTCCTCGAAAGCTACATATAAGGAA) and URA3-tec1-3UTR-R (ATACGTATGCGTATTTATGTACGAGATGTATGTATGTATGTAGACATTTAGT

TTTGCTGGCCGCATCTTC), a myc-Ura3-encoding DNA with *TEC1* UTR flanks was generated by PCR and used to transform a *ura3Δ0 myc-TEC1* strain to Ura+. These same primers were used to generate a *URA3* probe for northern-blot analyses. As a control, a *myc-URA3* strain (myc-Ura3 expressed from the *URA3* gene) was made. First, from a *URA3*+ strain, Ura3 protein-coding sequences were deleted and replaced with a NatMX4 cassette [6]. We used the resulting *ura3*Δ::*NatMX4*, rather than *ura3Δ0*, because the *ura3Δ0* deletion extends beyond the Ura3 protein-coding sequences. Next, using primers ura3_3MYC_F (TTCTTAACCCAACTGCACAGAACAAAAACCTGCAGGAAACGAAGATAAAT CATGAGGGAACAAAAGCTGG) and URA3-native3'_R (GCTCTAATTTGTGAGTTTAGTATACATGCATTTACTTATAATACAGTTTTT AGTTTTGCTGGCCGCATC) and genomic DNA from a *TEC1*::*myc-URA3* strain as the template, *myc-URA3* encoding DNA with primer-encoded *URA3* UTR flanks was amplified and used to transform a *ura3*Δ::*NatNX4* strain to Ura+.

Multicopy MPT5+ plasmid construction. Plasmid pG170 is a multicopy yeast plasmid bearing the *MPT5*+ gene. *MPT5* coding sequences, plus 3000 bp upstream and 500 bp downstream, were PCR-amplified from genomic DNA and cloned as an *Xma*I fragment (sites encoded in the PCR primers) into the *URA3* 2-micron vector plasmid pRS426 [5]. Several independent clones, including pG170, gave identical results in phenotype tests (data not shown).

MAPK-pathway reporter constructions. The fMAPK-pathway reporter construct consists of: a *Saccharomyces kluyveri HIS3*+ marker gene, in the opposite orientation from the reporter; an FRE element upstream of *CYC1* basal promoter sequences for polymerase binding; GFP(S65T) coding sequences; and an *ADH1* transcriptional terminator. FRE-CYC1 promoter sequences, plus an ATG, were PCR-amplified from pFRE(Ty1)-LacZ [7] using Pfu Turbo polymerase (Stratagene) and primers

FRE_CYC1_f1 (AAAAGATCTCATTCTTCTGTTTTGGAAGCT) and FRE_CYC1_r1 (AAATTAATTAACATTATTAATTTAGTGTGTGTATT). The product, with flanking primer-encoded *Bgl*II and *Pac*I sites, was digested with these enzymes and cloned into the *Bgl*II and *Pac*I sites of pFA6a-HIS3MX6-PGAL1-GFP [3], thereby replacing the *GAL* promoter with the FRE-CYC1 promoter and fusing the *CYC1* ATG in frame to GFP coding sequences. The resulting plasmid, pG195, was used as a PCR template to amplify, with primers FRE_CYC1_f2 (TATACTAAAAAATGAGCAGGCAAGATAAACGAAGGCAAAGGAATTCGAGCTCGTTTAAAC) and FRE_CYC1_r2 (TATATATATCGTATGCTGCAGCTTTAAATAATCGGTGTCAGGATCTGCCGGTAGAGGT), and integrate the entire construct by transformation at the site of *his3Δ0::hisG* to generate *his3Δ0::HIS3::FRE-GFP*. Note that the reverse primer sequence suggested by Longtine *et al.* [3] was not used because it would exclude the GFP stop codon and *ADH1* terminator. To make a PRE-GFP reporter, FRE sequences in plasmid pG195 were replaced with the triple Pheromone Response Element (PRE) sequences of the *PRM1* promoter [8]. Reverse complementary PRE-containing oligonucleotides, 3PREf (AAAAGATCTATATGTTTCAATACTGTTTCAATACTGTTTCAGAAGCTCGAGAAA) and 3PREr (TTTCTCGAGCTTCTGAAACAGTATTGAAACAGTATTGAAACATATAGATCTTTT), were hybridized and digested with *Bgl*II and *Xho*I. This fragment was cloned into pG195 DNA digested with the same enzymes to generate plasmid pG212. The FRE and PRE sequences of pG195 and pG212 were verified by sequencing. Using pG212 as a template, the *PRE-GFP* reporter was integrated into the genome as described above for the *FRE-GFP* reporter.

Immunoprecipitation and phosphatase treatment. Immunoprecipitation of N-terminally tagged Tec1 and Ste7 from an *mpt5*Δ strain with subsequent phosphatase treatment was done as described previously [9].

mRNA 3'-end mapping. The extents of the 3' UTRs of the *TEC1* and *STE7* mRNAs were determined using the Invitrogen 3′ RACE System according to the manufacturer's specifications. Total RNA was isolated from a wild-type strain grown under filamentous-form conditions for 10 hours, and used as the template for first-strand cDNA synthesis with the kit-provided AP primer. *TEC1* and *STE7* cDNA was PCR amplified with kit primer AUAP and the gene-specific primers TEC1_RACE (GAAAG TAATCCTGAGTTCAGTTCCA) or STE7-altern-F (ATAACGATACACCTGATGGC ATATT) respectively. A second, nested, PCR reaction used primer Tec1_fw_nested (*TTTGAT*CACCCCAGCATTACGAACA; extragenomic sequences in italics, *Bcl*I restriction site underlined) or STE7_RACE (CAAGATTACCCAAAGACCGTATCTA) along with kit primer AUAP. *TEC1*-specific PCR products were cut with *Bcl*I and *Sal*I, cloned into plasmid Bluescript KS+ cut with *Bam*HI and *Sal*I, and sequenced. *STE7*-specific PCR products were cloned as a *Cla*I, *Sal*I fragment into plasmid Bluescript KS+ and sequenced.

REFERENCES FOR SUPPORTING MATERIALS AND METHODS

1. Guthrie C, Fink GR (1991) Guide to Yeast Genetics and Molecular Biology. New York: Academic Press.
2. Drees BL, Thorsson V, Carter GW, Rives AW, Raymond MZ, et al. (2005) Derivation of genetic interaction networks from quantitative phenotype data. Genome Biol 6: R38.
3. Longtine MS, McKenzie A, 3rd, Demarini DJ, Shah NG, Wach A, et al. (1998) Additional modules for versatile and economical PCR-based gene deletion and modification in Saccharomyces cerevisiae. Yeast 14: 953-961.
4. Schneider BL, Seufert W, Steiner B, Yang QH, Futcher AB (1995) Use of polymerase chain reaction epitope tagging for protein tagging in Saccharomyces cerevisiae. Yeast 11: 1265-1274.
5. Christianson TW, Sikorski RS, Dante M, Shero JH, Hieter P (1992) Multifunctional yeast high-copy-number shuttle vectors. Gene 110: 119-122.
6. Goldstein AL, McCusker JH (1999) Three new dominant drug resistance cassettes for gene disruption in Saccharomyces cerevisiae. Yeast 15: 1541-1553.
7. Madhani HD, Fink GR (1997) Combinatorial control required for the specificity of yeast MAPK signaling. Science 275: 1314-1317.
8. Heiman MG, Walter P (2000) Prm1p, a pheromone-regulated multispanning membrane protein, facilitates plasma membrane fusion during yeast mating. J Cell Biol 151: 719-730.
9. Prinz S, Avila-Campillo I, Aldridge C, Srinivasan A, Dimitrov K, et al. (2004) Control of yeast filamentous-form growth by modules in an integrated molecular network. Genome Res 14: 380-390.
