# Supplementary figures and images for: Control of Signaling in a MAP-kinase Pathway by an RNA-Binding Protein
Source: PLoS One. 2007 Feb 28;2(2):e249. doi: 10.1371/journal.pone.0000249 (PMC1803019; doi:10.1371/journal.pone.0000249)

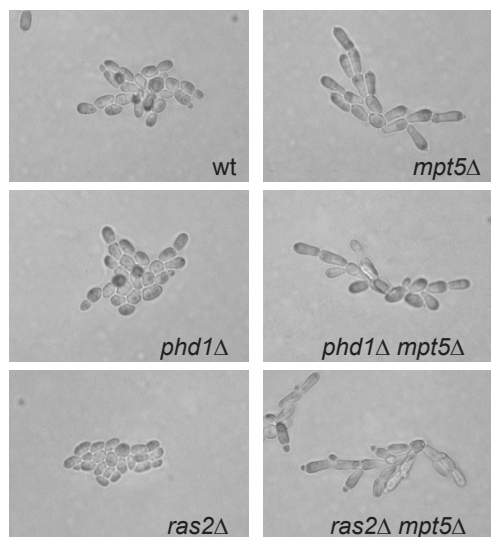

Supplement: Figure S1 — mpt5Δ phenotype requires neither PHD1 nor RAS2. Diploid yeast of the indicated genotypes were grown under filamentous-form conditions (SLAD agar) and microscopically imaged to show their filamentation phenotypes. (0.93 MB PDF) [file pone.0000249.s002.pdf]

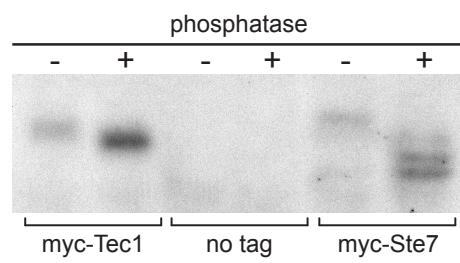

Supplement: Figure S2 — Phosphorylation of Tec1 and Ste7 in mpt5Δ strains. N-terminally tagged Tec1 or Ste7 protein, or a tagless control, was immunoprecipitated from an mpt5Δ strain with subsequent phosphatase treatment or mock treatment and analyzed by western blot. (0.92 MB PDF) [file pone.0000249.s003.pdf]

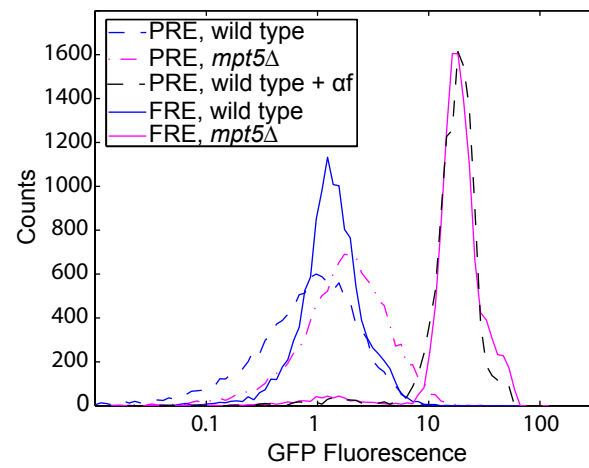

Supplement: Figure S3 — fMAPK and mMAPK pathway output in haploids. Haploid MATa strains with either a minimal filamentation-MAPK-pathway output reporter (FRE-GFP) or a mating-MAPK reporter (PRE-GFP) and the indicated genotypes were grown under yeast-form conditions in the absence of alpha factor and subjected to flow cytofluorometry. As a control, PRE-GFP output of an alpha-factor stimulated strain is shown. (0.52 MB PDF) [file pone.0000249.s004.pdf]
